# Successful Treatment of Osteitis Fibrosa Cystica from Primary Hyperparathyroidism

**DOI:** 10.1155/2012/145760

**Published:** 2012-09-04

**Authors:** Anthony M. Maina, Harry Kraus

**Affiliations:** Orthopaedic Department, AIC Kijabe Hospital, Kijabe 00220, Kenya

## Abstract

Osteitis Fibrosa Cystica (OFC) is defined as the classic skeletal manifestation of advanced primary hyperparathyroidism. With the increased detection by means of routine calcium screening, the clinical profile of primary hyperparathyroidism in Western countries has shifted from symptomatic disease to one with subtle or no specific symptoms (“asymptomatic” primary hyperparathyroidism). The authors describe a classical feature of advanced primary hyperparathyroidism due to a parathyroid adenoma and its successful treatment.

## 1. Introduction

Osteitis fibrosa cystica (OFC) is a skeletal disorder caused by a surplus of parathyroid hormone (PTH) from overactive parathyroid gland(s). This surplus stimulates the activity of osteoclasts, cells that breakdown bone. The overactivity of the parathyroid glands (primary hyperparathyroidism) can be triggered by parathyroid adenoma, hereditary factors, parathyroid carcinoma, or renal osteodystrophy. Majority of hyperparathyroidism is the result of parathyroid adenoma (80–85%) [1, 2]. The symptoms of the disease are the consequences of both the general softening of the bones and the excess calcium in the blood and include bone fractures, kidney stones, nausea, peptic ulcers, appetite loss, and weight loss—“bones, stones, abdominal groans and psychic overtones” [3]. Women are more often affected than men, and it occurs more frequently in the 5th and 6th decades. If it occurs in the younger (especially first decade), rule out hereditary causes—multiple endocrine neoplasia type I/IIa/IIb [4]. The serum calcium (8.4–10.2 mg/dL), PTH (15–65 pg/mL), and alkaline phosphatase (20–140 IU/L) are usually elevated. Plain radiographs distinctly show resorption, and the skull depicts the “ground glass”/“salt and pepper” appearance. The first bones to show X-ray features are the fingers. The cysts are lined by osteoclasts and sometimes blood pigments, which lend to the notion of “brown tumours”; such cysts can be identified with nuclear imaging combined with specific tracers, such as Sestamibi [5].

## 2. Case Report

**Figure 1:**
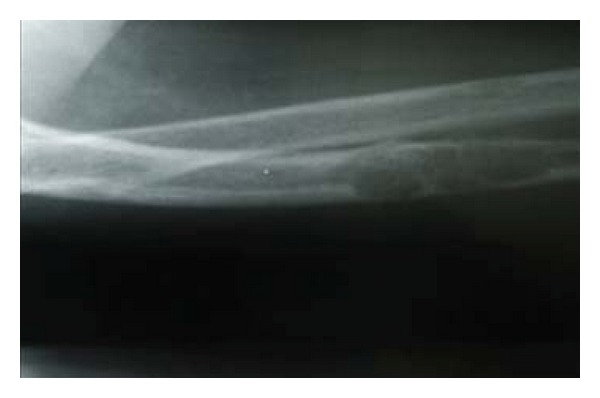
Lateral X-ray of the right radioulna showing osteitis fibrosa cystica lesion of ulna diaphysis with a pathologic fracture (before curettage).

**Figure 2:**
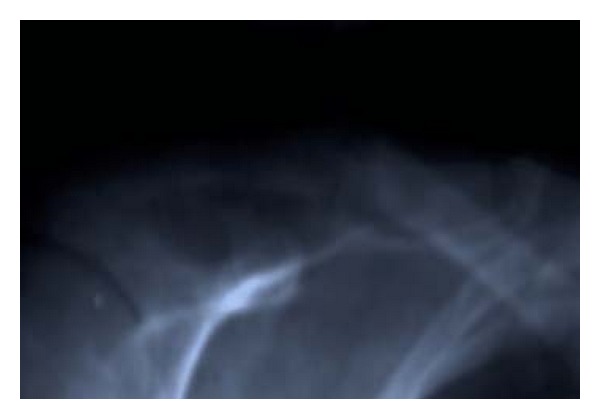
AP X-ray of the right clavicle showing osteitis fibrosa cystica lesion with a pathologic fracture (on admission).

**Figure 3:**
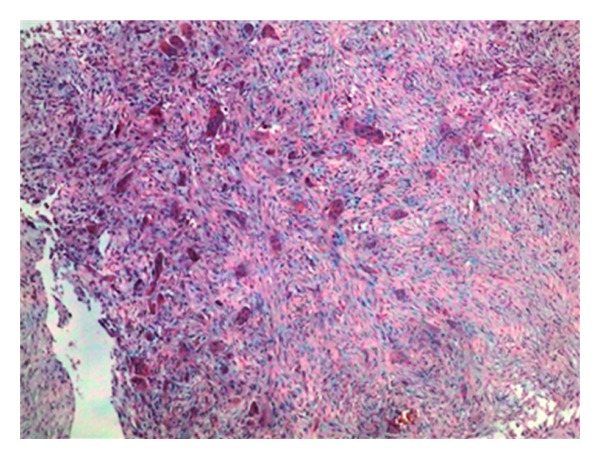
Histopathology slide showing brown tumour of hyperparathyroidism (multiple osteoclasts in a fibrous network); H & E stain, ×100 magnification.

**Figure 4:**
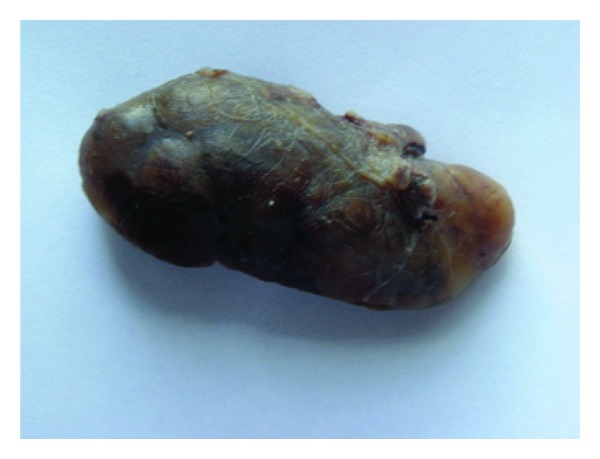
A gross specimen of one the parathyroid adenomas (whole).

**Figure 5:**
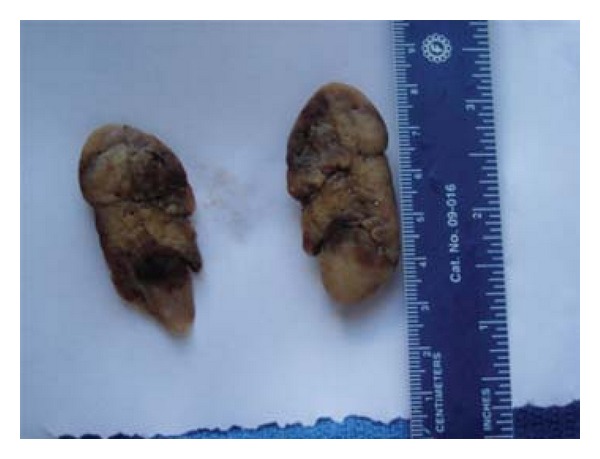
Gross specimen of a bisected parathyroid adenoma.

**Figure 6:**
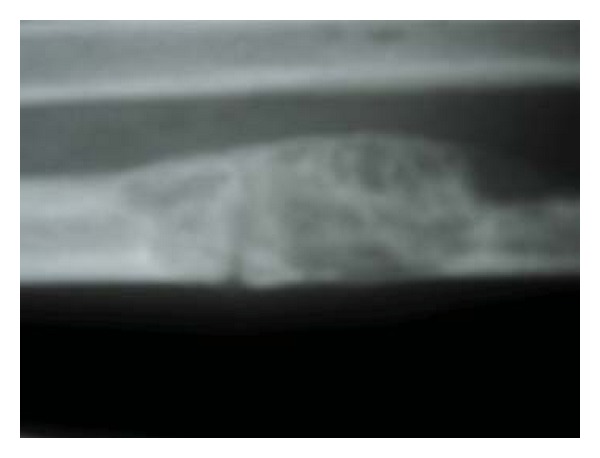
A lateral X-ray of the right ulna showing healing of the pathologic fracture in progress (8 weeks after curettage).

33-year-old female presented with a 5-year history of generalized body pain and inability to use her right upper limb after a fall 5 days prior to presentation. She also had fatigue and nausea. There was also a history of weight loss and she was easily upset during a conversation. Examination revealed a central neck mass that moved with swallowing but not discretely palpable. In addition, she had a tender right shoulder and forearm. Radiographs revealed generalized thinning of bone cortices and cystic lesions of the ulna and clavicle (both had pathologic fractures too) (Figures 1 and 2). On biochemistry, calcium and PTH levels were elevated, 10.6 mg/dL and 1203 pg/mL, respectively. Incision and curettage of the right ulna lesion were done, and histopathology reported it as brown tumour of hyperparathyroidism (Figure 3). This pointed to primary hyperparathyroidism due to overactivity of the parathyroid gland(s). Therefore, a neck exploration was warranted that yielded a 10-gram right superior pole parathyroid adenoma that was excised (Figures 4 and 5). Postoperatively, she was put on calcium carbonate 600 mg twice a day for 1 month and later tapered to 600 mg once a day for 2 weeks. Resultantly, the calcium levels normalized (9.3 mg/dL). The bone pain decreased and the cystic defects in the clavicle and ulna healed (Figure 6).

## 3. Discussion

First described in the nineteenth century, OFC is currently detected through a combination of blood testing, radiographs, and tissue sampling [3]. Before 1950, around a half of those diagnosed with hyperparathyroidism in the United States saw it progress to OFC, but with early identification techniques and improved treatment methods; instances of OFC in developed countries are increasingly rare. The high visibility of primary hyperparathyroidism in the population today marks a dramatic change from several generations ago when it was considered a rare disorder [3]. The increase in incidence is primarily due to widespread use of the autoanalyzer gratuitously providing serum calcium determination when a serum chemistry profile is ordered for another reason [3]. Where treatment is required, it entails addressing the underlying hyperparathyroidism before commencing long-term treatment for OFC. Depending on its cause and severity, this can range from hydration and exercise to surgical intervention [5]. Primary hyperparathyroidism is a curable disease with successful removal of the parathyroid adenoma [3]. This patient represents what was experienced in the developed countries before the 50s. The successful parathyroidectomy resulted in disease cure.

## 4. Conclusion

Parathyroidectomy has been shown to result in the reversal of bone resorption and complete regression of brown tumours [6, 7]. OFC is rare in developed countries due to early detections of hypercalcemia and its subsequent treatment [6, 8]. On the contrary, in developing countries where the multichannel autoanalyzer is not available or gratuitously used, OFC still exists [9]. This patient who was from the war-torn Somalia presented with classic OFC features. Successful diagnosis and the resultant parathyroidectomy yielded cure of disease.

## Abbreviations

OFC: Osteitis fibrosa cystica.
